# Hydrophobia and Pasteur’s Experiments

**Published:** 1885-12

**Authors:** 


### Hydrophobia and Pasteur's Experiments.

The wonderful announcement has recently been made that a cure has been found for hydrophobia, a disease not of frequent occurrence, but most terrible in its character and results. If a cure has been found, it is one of the most brilliant discoveries of modern therapeutics.

For years the great French savant, Louis Pasteur, has been working out the problem of ameliorating and preventing the communicable diseases of animals and man. Already his achievements have commanded the admiration of the whole scientific world; and those who are engaged in the industries of brewing, wine-growing, silkworm-raising, and poultry-keeping, will ever hold his name in grateful remembrance. Already he has won the veneration of our profession by demonstrating that Jenner's great discovery of vaccination was not an isolated and empirical fact, but that it was only a single member of a united family held together by the great scientific law, that disease may be abbreviated or prevented by the inoculation of a modified or attenuated virus of the same.

For over five years Pasteur has been conducting an extended series of experiments with hydrophobia, which have led to his latest and grandest achievement—the prevention and cure of this dread disease. The results of many of these experiments were given in his memorable address* delivered to a vast audience at the International Medical Congress at Copenhagen, August 11, 1884.

---

* *London Medical Times and Gazette*, August 23, 1884.

The main conclusions there enunciated were that hydrophobia possesses one poison only; that it had a period of incubation varying with the susceptibility of the subject and the methods of introduction into the system; that it never invades every part simultaneously, but is always developed in the brain, spinal cord, nerves, and salivary glands; that the virus is *always found*, when "death occurs as a natural result of the disease," at the "uppermost portion of the spinal cord," the "bulb," "which forms the point of transition between the cord and brain;" that the virus obtained from the "bulb" by macerating it in two or three times its bulk of sterilized liquid, will not fail to produce the disease when introduced into the arachnoid space through the trephined skull; that the incubation period is shorter when introduced from animal to animal of the same species; that the virulence is modified and weakened when transmitted through different species, monkeys, especially, diminishing it; that the test of degrees of virulence is determined with sufficient certainty by the duration of the incubating period, using the intracranial inoculation, and "a quantity of virus, which, although very weak, is more than sufficient to produce rabies;" that "the application of these facts yields a method of vaccinating dogs as a protection against rabies," taking, as "the starting point, one of the rabbits which have been inoculated from monkeys, to a sufficient degree that hypodermic or intravenous injection does not cause death."

Here Pasteur reached a point where he was enabled to prevent hydrophobia in dogs by the inoculation of a virus weakened by transmission through animals of different species, particularly the monkey. Nothing further of importance was given by him to the public until the latter part of October last, when he read a paper before the Paris Academy of Medicine, maintaining that he had discovered another method of weakening or attenuating the virus of hydrophobia, and that by a system of inoculation applied to the human subject, he had been able to render ineffective the bite of rabid dogs.

His method of attenuation is to transmit hydrophobia from rabbit to rabbit by intracranial inoculation, until, after some fifteen transmissions, the shortest period of incubation and the greatest degree of virulence have been attained. Then the spinal cord is taken from an animal dead of the disease, and it is suspended in a perfectly dry tube. Here it is kept, and the virulence of the poison is found to gradually diminish, until it at last disappears on about the fifteenth day. Several cords, varying from those that are fresh and extremely active to those that are stale and powerless, are kept for use. Dogs are rendered insusceptible to hydrophobia by inoculation first with the stale and powerless specimens, then with fresh and more active ones, and lastly, with the most fresh and virulent.

With these facts before him, Pasteur believed the same results might obtain in man as well, and he was led to make the experiment. The first opportunity of which he availed himself was presented in the case of an Alsatian boy nine years old, by the name of Joseph Meister, who was bitten in many places by a dog which, while alive, showed the symptoms common to the early stages of hydrophobia, and whose stomach after death was found stuffed with bits of straw, wood and refuse matter, indicating the depraved appetite characteristic of the disease. Twelve hours afterward, the wounds (probably not all of them) were cauterized with carbolic acid. On July 6, 1885, sixty hours after the infliction of the bites, and in the face of an almost certainty of hydrophobia, and doomed by Dr. Vulpian and Prof. Granger to death, Pasteur commenced his treatment. He began his inoculations by injecting material from a spinal cord fifteen days old — the virus here being very attenuated. The injections were repeated twice a day with virus of constantly increasing intensity till July 16th, when the spinal cord employed was quite fresh.

At the time of the reading of the paper, three months after the bites, Pasteur declared the boy radically cured. He continues in good health up to the present time.

Pasteur has, on a second occasion, repeated the same method of treatment upon a young shepherd, who, in defending other boys, was cruelly bitten by a mad dog, which he killed on the spot. This patient is also reported, by Pasteur, cured.

The validity of these "cures" must, of course, be left to the test of time. There are several considerations that must hold the judgment in abeyance. It is not absolutely certain that the dog which bit young Meister had hydrophobia, as he was immediately killed, although the symptoms and post-mortem signs indicated that he had the disease. Again, the boy's wounds were cauterized, but it was twelve hours after the bites, and it appears that all were not touched. Furthermore, the period of incubation in hydrophobia has been known to extend over several months, and indeed several years, one case having been reported to have occurred five years after the bite. On the other hand, waiving a long period of incubation, if the two boys were not cured of hydrophobia by Pasteur, what prevented his inoculations producing the disease in them?

Although these experiments, therefore, lack the elements of undoubted conclusiveness, yet they mark a progress in the history of therapeutical research of which the medical profession may justly be proud, and they deserve the enthusiastic applause of a large and expectant audience, anxiously awaiting the next scene of the panorama.

---

*Le Progrès Médical* for November 7th is a student's number, consisting of sixty-two quarto pages. The present status of medical education in France and thirty other countries is accurately described. With the exception of China and Turkey, the requirements for a medical degree are lower in the United States than in any other country. We cannot give in full the French opinion of American medical schools, but the following may serve as an example: "American students are always in haste to obtain their diplomas. They make an exact estimate of their time and money, and go to the school which will give the most with the least expenditure. The requirements for admission are nil, or else consist of a sort of examination in grammar. The examination for graduation furnishes no evidence that the student is qualified to practice. If a school attempts to raise its standard of requirements, the students immediately leave for those which are less exacting, as was the case with Bellevue Medical College in New York. The only school which requires a thorough medical education, before graduation, is Johns Hopkins University of Baltimore, which is well endowed. Some other schools, however, are attempting to improve the condition of medical education by adding a third course, the attendance being advised, but not always required. A few also require an entrance examination in Latin and the natural sciences. As a rule, the courses of medical studies in the United States are imperfect and rudimentary. Schools are very numerous in the United States; most of the large towns have at least one, while the principal cities, such as New York, Philadelphia, Boston, Chicago, and San Francisco, have three or four each, without counting *homœopathic*, *eclectic* and *female medical colleges*."

The only medical schools in America which are considered worthy of mention are: Johns Hopkins University; Harvard; University of Pennsylvania; Jefferson; College of Physicians and Surgeons of New York; Bellevue; University of New York; University of Louisiana; New York Past-Graduate Medical College, and "*the modest University of Niagara, located at Buffalo, and which, during the scholastic year 1883–84, had thirty students.*"

The period of medical study required before the final examination in other countries is as follows: France, four years; England, three years and nine months; Germany, four years and six months; Austria, five years; Denmark, six or seven years; Norway, five years; Sweden, eight or nine years; Belgium, seven years; Italy, five or six years; Portugal, Russia and Uruguay, each five years; Mexico, Chili, U. S. Colombia and Argentine Republic, each six years; Peru and Venezuela, seven years; Japan, four years.

